# Thin and in situ melanoma: an update for the dermatologist^؆^

**DOI:** 10.1016/j.abd.2026.501349

**Published:** 2026-04-30

**Authors:** João Renato Vianna Gontijo, Flávia Vasques Bittencourt, Jacob H. Nelson, Lucas Campos Garcia, Sancy A. Leachman

**Affiliations:** a Department of Internal Medicine, Faculty of Medicine, Universidade Federal de Minas Gerais, Belo Horizonte, MG, Brazil; b Service of Dermatology, Rede Mater Dei de Saúde, Belo Horizonte, MG, Brazil; c Department of Dermatology, School of Medicine, Oregon Health and Science University, Portland, OR, United States of America; d Department of Dermatology, School of Medicine, University of Utah, Salt Lake City, UT, United States of America

**Keywords:** Melanoma, Neoplasm metastasis, Prognosis, Recurrence

## Abstract

**Background:**

Thin melanoma (TM, ≤1.0 mm Breslow thickness) and Melanoma In Situ (MIS) constitute the majority of melanoma diagnoses worldwide and are responsible for melanoma-related deaths in these early-stage tumors. Despite their favorable prognosis, MIS and TM represent an opportunity for improving patient outcomes through early detection, accurate risk stratification, and long-term surveillance for metastasis and new skin neoplasms.

**Objective:**

Provide an update of current evidence regarding epidemiology, risk factors, prognostic indicators, genetic background, and clinical management of MIS and TM.

**Methods:**

A comprehensive review of the literature and international guidelines was conducted, integrating epidemiologic data, clinical prognostic parameters, and molecular insights relevant to MIS and TM.

**Results:**

MIS and TM account for over 80% of all melanomas, with increasing incidence and relatively stable mortality rates. Prognosis is primarily determined by Breslow depth and ulceration, while factors such as mitotic rate, anatomic site, and age further refine risk assessment. Genetic alterations contribute to tumorigenesis but are not yet integrated into routine management. Long-term dermatological surveillance is needed, as new neoplasms, recurrence, and metastasis can develop during follow-up.

**Conclusions:**

MIS and TM are increasingly diagnosed, and dermatologists need to be a part of early detection, multidisciplinary management, and lifelong surveillance, which remain the cornerstone of reducing melanoma-related mortality.

**Study limitations:**

The substantial heterogeneity among the included studies limits direct comparison and quantitative synthesis of the available data.

## Introduction

In recent years, advances in the genetic characterization of melanomas, early diagnosis, and new treatments have changed the perspective and knowledge of this tumor. Even in the absence of a defined model of melanoma evolution, the clinical and epidemiological importance of Melanoma In Situ (MIS) and Thin Melanoma (TM) (Breslow thickness ≤1.0 mm), which comprises the vast majority of cases, is well established.

Increased use of dermoscopy, translation of novel technologies into clinical practice, improved melanoma awareness in clinical practice and public health campaigns all contribute to earlier diagnosis of skin tumors, leading to an increasing number of patients with MIS and TM being diagnosed and managed by dermatologists.

Progression of melanoma to invasive and metastatic disease has a substantial impact on public health, as these stages account for most new diagnoses and are responsible for approximately one-third of melanoma-related deaths. Moreover, MIS and TM are important risk factors for the development of subsequent primary melanomas and other cutaneous neoplasms, underscoring the necessity of long-term dermatologic surveillance.

## Epidemiology

Unlike most other tumors, the overall global incidence of cutaneous melanoma has been steadily increasing in recent decades, with mortality stabilizing over the years.^1^ Despite being a less frequent skin tumor, its lethality is responsible for almost 73% of all deaths from skin cancers.^2^, ^3^

**Fig. 1:**
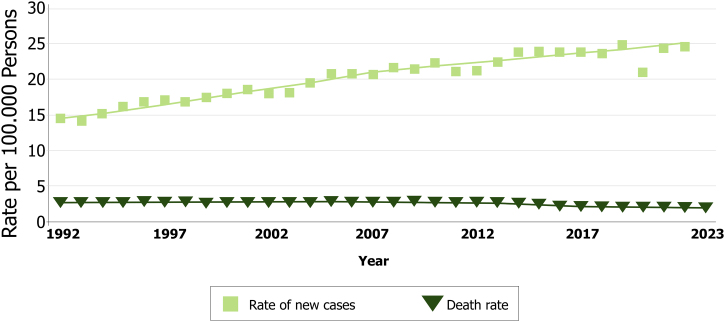
Rate of new melanoma cases and deaths per 100,000 persons in the United States of America over the years. Reprinted with permission from Cancer Stat Facts: Melanoma of the Skin. Surveillance, Epidemiology, and End Results (SEER) program.

There is debate about the cause of increased melanoma incidence and its reliability, since the increase is only observed in cutaneous melanomas and is not accompanied by increased mortality (Fig. 1).^4^, ^5^, ^6^ Some authors hypothesize that the increase in incidence is primarily driven by an increased tendency for pathologists to diagnose melanoma in lesions that were previously considered to be only atypical or benign.^7^ Overdiagnosis of undoubted melanoma patients that died from other pathologies and were exempt from autopsy is also a confounding factor, since their mortality is often attributed to melanoma. However, it is more likely that this rise in incidence is multifactorial, including greater exposure to Ultraviolet (UV) radiation, population aging, improvement of surveillance services that record tumors, definition and standardization of histopathological criteria, early detection campaigns, and the use of diagnostic tools such as dermoscopy, which has refined the diagnostic accuracy of melanoma.^5^

This discrepancy between rising melanoma incidence and relatively stable or declining mortality rates should not deter individualized medical decision-making regarding patient treatment and follow-up. Similarly, patient education and early detection efforts should not be deprioritized, as they can significantly influence disease awareness, facilitate earlier diagnosis, and ultimately improve prognosis.

The Surveillance, Epidemiology, and End Results (SEER) registry reports that in 2020 in the USA, the age-adjusted incidence rates for MIS were 18.39/100,000 and 11.32/100,000 for TM.^8^, ^9^ The *Instituto Nacional do Câncer* (INCA) estimated 8,980 new cases of melanoma in Brazil for 2023‒2025. Following a worldwide trend, the incidence of melanoma is higher in men, with 4640 new cases and 4340 in women, with a national incidence (across all stages) of 4.13 cases per 100,000 persons and a higher incidence in the south of the country. In 2020, there were 1923 melanoma-related deaths in Brazil, comprising 1120 deaths among men and 803 among women.^10^

Currently, most of the melanomas diagnosed are MIS and TM, globally and in the USA, accounting for 83% of all cases.^9^ Although they usually have a good prognosis, a small percentage of these patients will have disease progression, and since they are very numerous, MIS and TM melanomas are responsible for 30% of all melanoma deaths.^11^

Recently, global and national databases have improved the registration process substantially, including the implementation of automatic reporting and staging verification. Identification of late-stage melanomas is more reliable due to the use of hospital, regional pathology lab, and death records, whereas early-stage melanomas are likely to be relatively more difficult for the registry to document. This is likely responsible for some of the observed increasing incidence of melanoma and possibly contributes to the disparity between incidence and mortality increases. There is a significant possibility that melanoma cases may still be underreported, leading to errors in large databases, which could result in the underrepresentation of these tumors.

## Risk and prognostic factors

New insights into melanoma risk and prognostic factors have been published in recent years. This is important so that patient awareness and better risk stratification can be achieved. Prognostic factors are related to disease progression and are constantly being assessed and updated by the American Joint Committee on Cancer (AJCC) and the National Comprehensive Cancer Network (NCCN).

**Fig. 2:**
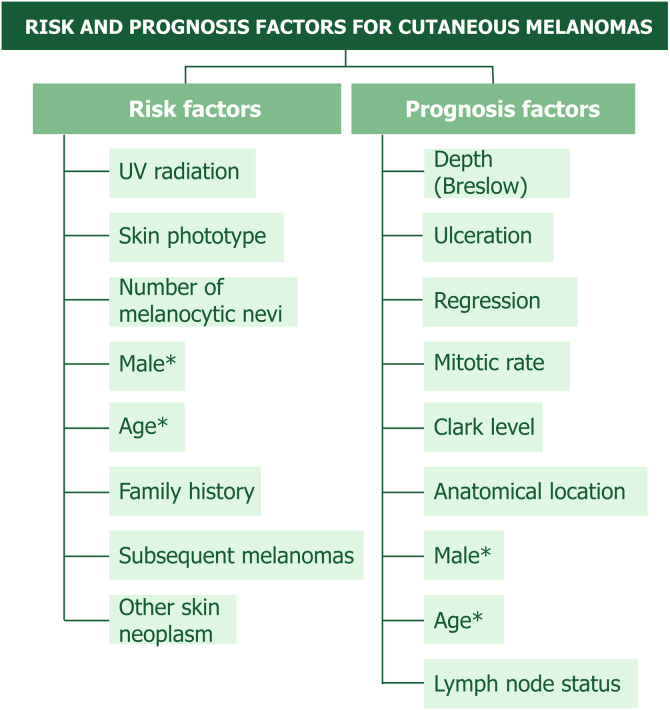
Well stablished risk and prognostic factor in patients with cutaneous melanoma. * Some features such as age and sex are mutual factors. Pigmentation phenotype includes lightly pigmented skin, hair, eyes, red hair and freckles as risk factors.

Few studies have established specific MIS and TM risk factors. Fig. 2 provides a schematic view of these factors so they can be easily addressed during patient consultation and counseling.

### UV radiation, skin phototype and melanocytic nevi

Exposure to UV radiation is the most common environmental risk factor for skin tumors. The mutations caused by UV radiation can be considered a pathogenic factor, acting from neogenesis to advanced stages of melanoma. Individuals’ phenotypes are controlled by the ratio of eumelanin and pheomelanin. Eumelanin provides protection against UV-induced DNA damage and is almost not present in red-haired and Caucasian individuals, who have more pheomelanin and have a greater tendency to develop skin cancers.^12^

Risk of melanoma is correlated with the number of sunburns that a patient has suffered, which are more common in individuals with lightly pigmented phototypes. Individuals with dark skin phototypes are not exempt from melanomas, which occur preferentially in acral topography, with a worse prognosis, often with advanced disease. Recent mutational data have shown that acral melanomas have a low mutational burden, suggesting that it is not a UV-induced malignancy.^13^ The risk of individuals with lightly pigmented phototypes developing melanoma is 10 times higher when compared to individuals with dark skin.^14^

Evidence suggests that the number of Melanocytic Nevi (MN) is more important as an individual risk marker for the development of melanoma than as precursor lesions. About one-third of melanomas originate from pre-existing nevi, occurring most commonly on the trunk of young patients, while 70% are *de novo*.^15^ These data indicate that most melanomas do not originate from the malignant transformation of nevus cells. A challenge in the MN approach is the differential diagnosis with MIS, especially in dysplastic nevi with severe atypia.

### Anatomical location, sex and age

Melanomas located on the head and neck deserve particular care due to their worse prognosis. They occur in the elderly, and their frequency is considered high (up to 26.7%) for an area that corresponds to only 9% of the body surface.^16^, ^17^, ^18^

The trunk is the most affected site in men (41.5%) and the lower limbs (32.7%) in women.^19^ Previous studies reveal that a worse prognosis is expected in male patients, increased age, and sites such as the head and neck or trunk.^20^, ^21^, ^22^ Men generally have a higher mean age (56-years) at the time of diagnosis than women (52-years).^19^

### Family history, subsequent melanomas and other skin neoplasms

Family History (FH) of melanoma is a well-defined risk factor. Wei et al. followed 216,115 individuals, finding a 74% increased risk of melanoma (Hazard Ratio [HR = 1.74]) when compared with those without FH. Hereditary melanomas have an increased risk of cancers in other organs, such as the breast, pancreas, or central nervous system.^23^, ^24^

A prior history of any melanoma should be considered a high-risk factor for cutaneous melanoma, with 1% to 8% of these patients developing multiple melanomas.^14^ On follow-up, 18.7% of MIS and TM patients developed a second melanoma.^25^ The subsequent tumor is usually thinner than the first, and its risk is higher in patients with fair skin and hair and an increased number of nevi.^19^

Individuals with a FH also have a 22% increased risk (HR = 1.22) of Squamous Cell Carcinoma (SCC), 27% (HR = 1.27) for Basal Cell Carcinoma (BCC), and an increased risk of melanomas on the trunk in both sexes and SCC on the extremities in women.^26^

In a meta-analysis, the lifetime risk of developing secondary skin tumors, after a primary melanoma, was 3.8% for a new melanoma, 2.8% for BCC and 1% for SCC. The calculated 20-year cumulative risk was 5.4% for a second melanoma, 14% for BCC, and 4% for SCC. Although the analyses by subgroups and continents show substantial differences, the previous history of melanoma is a strong predictive factor for the development of a subsequent melanoma (approximately 10-fold increase in RR).^27^

As the lifespan of patients with melanoma has increased with new treatments, the likelihood of new melanomas and SCC/BCC also increases, and greater surveillance is needed in this group.^25^, ^28^

### Breslow

Breslow thickness (depth or index) represents the measurement in millimeters from the granulosa layer of the epidermis to the maximum depth of tumor invasion. This measurement is the most important prognostic factor for metastasis used by the AJCC for staging.^29^, ^30^

New data indicate a “breakpoint” in 0.7 to 0.8 mm for the survival of T1 patients; this subgroup of TM should be assessed for their high risk of disease progression and Lymph Node Biopsy (LNB) should be considered in the multidisciplinary tumor board.^31^ Invasive melanomas with Breslow depth ≥0.8 mm have a 1.7 hazard ratio of worse survival than patients with <0.8 mm.^30^ TM patients with Breslow thickness between 0.8 and 1 mm also have a six-fold risk for progression to death, and the same six-fold risk for head and neck localization, when compared to tumors <0.8 mm. Melanomas with higher Breslow thickness should be monitored more frequently, especially if other associated risk factors are present.^25^, ^32^, ^33^

### Ulceration

Besides Breslow depth, the presence of ulceration in the primary tumor is the most important pathological prognostic indicator in melanoma, being associated with aggressive disease and risk of Lymph Node (LN) metastasis.^30^, ^34^, ^35^, ^36^ This is reflected by upstaging these patients in the AJCC when ulceration is present. MIS does not have any ulceration, and it is rare in T1 patients.

### Regression

Patients with invasive tumors may present with partial regression on pathology, a phenomenon that may represent an immunological response to the melanoma. Macroscopically, this may present as pink, grayish, hypopigmented or depigmented areas.^37^, ^38^ The influence of regression on prognosis remains unknown, with some studies considering it to be a negative prognostic factor, because of the difficulty in accurately assessing Breslow thickness in regressed areas.^39^ This phenomenon was associated in some studies with a better prognosis, since effective activation of the host immune system against melanoma cells is likely its basis.^40^ Regression is usually measured based on changes that are present in the dermis, which makes this factor inappropriate for MIS.^38^

### Mitotic rate

Despite being removed from the last AJCC 8^th^ staging edition, this index remains an important prognostic factor in most studies. It is considered an independent prognostic factor for LN positivity in TM, along with Breslow thickness in several studies.^29^, ^35^, ^41^, ^42^ However, the reproducibility of this risk factor, including interobserver variability and conflicting data on the number of mitoses that would be considered the threshold to become a factor of worse prognosis, makes it harder to standardize.

### Clark level

For decades, Clark's levels of invasion have been used in conjunction with Breslow thickness for staging and classification in past AJCC editions. The challenge associated with reproducibility in measurements of Clark’s levels among observers has precipitated the abandonment of this parameter in recent years.^43^

Although it is not used for staging in the AJCC 8^th^ edition, the Clark level is a well-established prognostic factor and correlates with increased mortality in most studies.^33^, ^35^, ^39^ Clark level is important for TM evaluation and risk stratification and is a part of a complete pathological report.

## Genetic aspects

Genetic profiling of melanomas will most likely provide missing information on tumor progression, therapeutic targets and personal staging in the upcoming years. It is important for the classification and identification of mutations in different populations, stages, and anatomical sites in an academic scenario (Table 1).^24^, ^44^, ^45^, ^46^

**Table 1:** Mutations related to cutaneous melanomas, syndrome types, and associated cancers.

Gene mutation	Syndrome type	Syndrome name	Associated cancers
CDKN2A	Dominant	FAMMM, FMPC	Cutaneous melanoma, pancreatic cancer, CNS tumors (astrocytoma)
CDK4	Dominant	Familial Melanoma Syndrome	Cutaneous melanoma
BAP1	Dominant	BAP1 Tumor Predisposition Syndrome	Uveal melanoma, cutaneous melanoma, mesothelioma, renal cell carcinoma, atypical Spitz tumors
MITF (E318K)	Dominant	Familial Melanoma	Cutaneous melanoma, renal cell carcinoma
POT1	Dominant	Familial Melanoma	Cutaneous melanoma, glioma, angiosarcoma, leukemia
TERT	Dominant	Familial Melanoma (emerging)	Cutaneous melanoma, various cancers
NF1	Subordinate	Neurofibromatosis Type 1	Nerve sheath tumors, glioma, breast cancer, pheochromocytoma, melanoma risk increased
BRCA1/2	Subordinate	Hereditary Breast and Ovarian Cancer Syndrome	Breast, ovarian, prostate, pancreatic, melanoma risk increased
PTEN	Subordinate	Cowden Syndrome	Breast, thyroid, endometrial, colon, melanoma risk increased
TP53	Subordinate	Li-Fraumeni Syndrome	Breast, sarcoma, brain tumors, adrenocortical carcinoma, melanoma risk increased
ATM	Subordinate	ATM-associated Hereditary Cancer Syndrome	Breast, pancreatic, melanoma risk increased
CHEK2	Subordinate	CHEK2-associated Hereditary Cancer Syndrome	Breast, colon, prostate, melanoma risk increased
MLH1, MSH2, MSH6, PMS2	Subordinate	Lynch Syndrome	Colorectal, endometrial, ovarian, stomach, hepatobiliary, urinary tract, melanoma risk increased
PALB2	Subordinate	PALB2-associated Hereditary Cancer Syndrome	Breast, pancreatic, melanoma risk increased
APC	Subordinate	Familial Adenomatous Polyposis (FAP)	Colorectal, hepatoblastoma, medulloblastoma, thyroid, melanoma risk increased
TERF2IP, ACD	Dominant	Emerging familial melanoma syndromes	Cutaneous melanoma, limited data
NBN	Subordinate	NBN-associated Cancer Syndrome	Breast, prostate, melanoma risk increased
RAD50	Subordinate	RAD50-associated Cancer Syndrome	Breast, ovarian, melanoma risk increased
SMARCA4	Subordinate	SCCOHT	Ovarian, melanoma risk suggested

Dominant syndromes ‒ melanoma is the major type of cancer in this syndrome. Subordinate syndromes ‒ melanoma risk is elevated, however it is not the dominant cancer type. CNS, Central Nervous System; FAMMM, Familial Atypical Multiple Mole Melanoma syndrome; FMPC, Familial Melanoma and Pancreatic Cancer syndrome; SCCOHT, Small Cell Carcinoma of the Ovary Hypercalcemic Type.

Somatic genetic mutations in early melanomas are distinct and necessary for tumorigenesis and disease progression. The most common oncogenic mutations are *BRAF* (commonly *V600E*), *NRAS*, *Kit,* especially in acral and mucosal subtypes.^46^, ^47^, ^48^

*BRAF-V600E* can be found in 28% of lethal TM patients, as this could be a potential marker, probably associated with other mutations and a treatment target in the future.^49^ They are also found in primary, metastatic, and melanoma cell linages, suggesting that they occur before tumor progression and spread and remain at a constant incidence during progression.^12^ *BRAF* mutation can occur early and be found in more than 80% of patients with common acquired MN and dysplastic nevus, and is considered a benign feature of nevi formation. Since these pigmented lesions rarely progress to melanoma, it can be concluded that other mutations and additional genetic changes are required for tumor progression.^12^, ^49^, ^50^

Germline mutations predispose individuals to melanoma due to hereditary predisposition and syndromes. Multiple genes such as *CDKN2A*, *CDK4*, *BAP1*, *POT1* and *MITF* are correlated to melanoma-dominant syndromes. Subordinate syndromes are associated with *BRCA1/2, PTEN* and *TP53* mutations and contribute to an increased melanoma risk and other cancers (e.g., pancreatic, astrocytoma, breast, colon, ovarian, prostate, BAP1 syndrome) in an individual context.^21^, ^24^, ^46^

About 10% of melanomas are associated with germline mutations, and these can increase the risk of melanoma by four to 100-times.^23^ Progression to metastatic disease is probably due to a combination of mutations and the individual immune system. It has been associated with mutations in the gene *PTEN* or *TP53,* there is a lack of studies in MIS and TM patients.^50^, ^51^ Genetic testing in high-risk individuals with multiple primary melanomas or FH of melanoma and other cancers is available for genetic counseling.

Environmental risk factors such as UV radiation from early and intermittent sun exposure, and individual factors (lightly pigmented skin, hair, eyes, red hair and freckles) tend to result in a high mutational burden (>10 mutations per megabase), with a high number of mutations typical of UV damage.^50^ This environmental exposure predisposes to *BRAF*-driven melanomas, usually in younger patients, on non-sun damage on the skin (e.g., trunk) and melanoma of the superficial extensive type.^50^, ^52^ Chronic sun exposure, on the other hand, is associated with mutations in *NRAS*, unrelated to the MN number.^50^

Gene Expression Profiling (GEP) represents an emerging adjunctive tool for the diagnostic and prognostic evaluation of cutaneous melanoma, though its integration into routine clinical practice remains under active investigation. They can aid the diagnosis of challenging melanocytic, however, GEP results should not supersede established histopathological criteria in guiding critical management decisions such as LNB or imaging surveillance strategies.^45^, ^46^

Current knowledge about the genetic alterations that participate in the development of initial MIS and TM is insufficient, and genetic testing should not be performed routinely. Different combinations of mutations have been found, and new genes discovery increases the number of possible genetic combinations. Mutations in high-penetrance genes, such as *CDKN2A*, *CDK4,* and *BAP1*, confer a 60% to 90% lifetime risk of melanoma.^23^ The future use of mutation biomarkers for risk stratification, choice of imaging, LNB, and adjuvant therapy is promising, but there is still no consensus for its use, requiring further studies.^53^, ^54^

Melanoma Prevention Working Group guidelines state that genetic testing should be analyzed as continuous variables to avoid low- and high-risk dichotomous interpretations that may have no biological significance. Results of these genetic profiles should always be evaluated and compared with established prognostic factors and by the risk stratification of the AJCC, and there are not yet sufficient data for their routine use.^54^

Currently, commercial use of genetic testing on different platforms can aid pathologists in challenging melanocytic lesions.^44^ There are no specific guidelines for genetic analysis in MIS or TM for risk stratification, treatment, or follow-up. It is the authors’ opinion that genetic profiling of these initial tumors could contribute to the future, so treatment and follow-up can be tailored to each patient.

## Diagnosis and treatment

Diagnosis should be made by clinical and dermoscopic evaluation, followed by anatomopathological examination. Dermoscopy allows the magnification of structures not visible to the naked eye, in the superficial epidermis and dermis, and is mandatory for dermatologists and health professionals caring for patients with melanocytic lesions and tumors.

The ABCD rule for the clinical diagnosis of melanoma, described in the 1980s, was a milestone for its earlier detection, especially considering that until then, large and ulcerated tumors were common.^55^ Later, the addition of the letter “E” to the ABCD acronym ‒ indicating “evolution” or “change” ‒ further refined melanoma diagnosis.^56^

This dynamic behavior of the lesion may occasionally be the only indication of the tumor, facilitating even earlier diagnoses, particularly in initial melanomas that may not exhibit a striking ABCD criteria. In the 1990s, dermoscopy improved the accuracy of melanoma diagnosis by more than 30%, revolutionizing the approach to these cutaneous tumors.^57^ This technique has allowed the identification of increasingly early melanomas, including melanomas that do not resemble typical ones.^58^

**Fig. 3:**
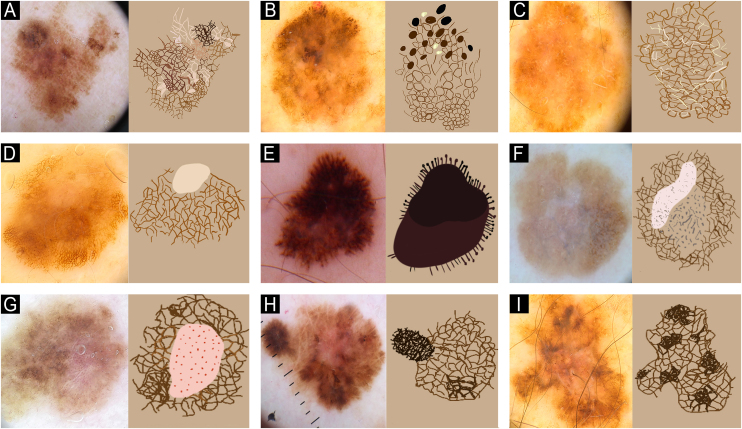
Dermoscopy features and their schematic features in MIS and TM. (A) Atypical, pigmented network. (B) Irregular globules and dots. (C) Negative pigmented network. (D) Peripheral tan and structureless areas. (E) Irregular radial streaks. (F) Regression. (G) Dotted vessels. (H) Dermoscopic islands. (I) Irregular pigmentation.

The main dermoscopic findings in MIS and TM (Fig. 3), include an irregular pigmented network, negative network, irregular globules and dots, radial streaks, irregular pigmentation, structureless areas, and dermoscopic islands (well-circumscribed areas showing a uniform dermoscopic pattern that differs from the rest of the pigmented lesion).

Photographic follow-up with total body mapping and digital dermoscopy has also contributed to earlier melanoma diagnoses while avoiding unnecessary removal of MN. It is plausible that this trend toward earlier diagnoses will continue to grow, especially with the implementation of artificial intelligence in dermatology.^59^

Initial biopsy should be, whenever possible, excisional, with minimal margins (1 to 3 mm), for complete pathological evaluation of the lesion and removing the least amount of unaffected skin, to avoid alteration in the local lymphatic drainage, with the longest axis in the same direction/parallel to lymphatic vessels.^46^

Special sites with aesthetic or functional impact, such as the face, extremities, and genitalia, where initial complete resection may lead to mutilation, incisional or punch biopsy may be performed and guided by dermoscopy. Confocal microscopy can aid in diagnosing challenging lesions and in guiding biopsies, especially in the face.^60^

Diagnostic challenges in MIS may arise from heterogeneity across histologic sections and overlap with severely atypical dysplastic nevi, which can promote inter-observer variability when dermatopathologists rely only on Hematoxylin-Eosin (HE) staining. This is relevant in melanocytic lesions with architectural disorder and cytologic atypia, where the differentiation between MIS and dysplastic nevi is uncertain even for experienced dermatopathologists.^15^, ^38^ Current diagnostic guidelines emphasize that Immunohistochemical (IHC) stains are not essential for the microscopic diagnosis of melanoma and should be reserved for selected cases in which morphology on HE is insufficient for the diagnosis; IHC should be used to support, rather than supplant, the primary histopathologic assessment.^24^, ^45^, ^46^, ^61^^,^^62^

IHC can be helpful in challenging MIS and TM cases where standard HE examination is uncertain or when dermo-epidermal junction architecture and cytologic features overlap with benign or atypical melanocytic proliferations. IHC panels include markers such as S-100, SOX10, Melan-A/MART-1, and HMB-45 that enhance melanocytic lineage identification and can identify deeper dermal invasion that may be underestimated on HE. IHC markers can have false positives or complicate interpretation in heavily pigmented lesions.^63^

Molecular diagnostic platforms, such as gene-expression-based assays, DNA-based sequencing, cytogenetic analyses, and copy-number assessment, can aid in selected cases of diagnostically challenging melanocytic tumors. Gene-expression assays evaluate the transcriptional profile of tumor cells to distinguish benign from malignant melanocytic proliferations and estimate metastatic risk in specific settings. DNA-based sequencing techniques identify somatic mutations in oncogenic pathways (e.g., *BRAF*, *NRAS*, *KIT*), providing insight into tumor genesis and potential therapeutic targets. Cytogenetic and copy-number techniques detect chromosomal gains, losses, or structural rearrangements that are more frequently associated with melanoma than with benign nevi.^45^, ^50^, ^54^, ^64^ These techniques also should not supersede established histopathologic criteria for diagnosis, management, and staging. Such methods have not yet been incorporated into routine clinical practice, they require further validation before their application in risk stratification and melanoma management.^24^, ^44^, ^45^

Cutaneous melanomas are classified according to their growth pattern, clinical and histopathological characteristics into four subtypes of invasive melanomas: Superficial Spreading (SS), Nodular Melanoma (NM), Acral Lentiginous (AL) and Lentigo Maligna Melanoma (LMM).^37^, ^65^ Lentigo Maligna (LM) is a subtype of MIS, which is slow growing and can evolve into an invasive component (LMM). These subtypes are not included as prognostic factors by the AJCC.^65^

After histological confirmation of melanoma, definitive surgical excision of scar tissue or residual lesion, along with adjacent tissue, should be planned and performed. NCCN 2025 guidelines for surgical margins of this definitive excision should be based on Breslow thickness (Table 2), and margins greater than 2 cm had no impact on Local Recurrence (LR) and survival.^46^ Whenever possible, the largest margin according to the Breslow thickness of the tumor should be performed, respecting the maximum value of 2 cm.^62^, ^66^

**Table 2:** Recommended margins for the surgical treatment of melanomas.

Tumor thickness	Surgical margin
*In situ*	0.5 – 1cm
≤1.00 mm	1 cm
>1.00 to 2.00 mm	1 ‒ 2 cm
>2.00 – 4.0 mm	2 cm

Surgery with intraoperative margin control (e.g., modified Mohs) associated with the use of IHC markers has been used in some countries with similar survival rates to standard surgery.^67^ Regular frozen sections without IHC can undergo artifact alterations, making the correct assessment of melanocytic lesions challenging, and should not be performed according to NCCN guidelines.^46^

LNB should not be performed in MIS. T1b melanoma (Breslow depth < 0.8 mm with ulceration or 0.8–1 mm with or without ulceration) should be assessed for LNB as a shared decision and discussed in multidisciplinary tumor boards.^46^, ^68^

LNB remains a crucial factor in the staging of patients and an important prognostic factor and predictor of survival.^69^ It remains the most sensitive and specific test to identify occult metastasis in LN, but it should not be routinely performed in TM.^70^ Complete LN dissection should not be performed since it does not impact patient survival.^71^

For greater uniformity, most studies use the AJCC system, staging tumors as in situ, according to Breslow thickness, LN involvement, and presence of metastasis (Table 3). MIS, by definition, do not exceed the basal layer and do not have Breslow depth, being staged as Tis. Accurate staging of patients by a dermatologist is mandatory so they can receive proper treatment, follow-up, and imaging when necessary.

**Table 3:** American Joint Committee on Cancer staging system for stage I and II patients.

T	Definition of the primary tumor	N	M	Clinical stage	Pathological stage
Tis	Melanoma *in situ*	0	0	0	0
T1a	< 0.8 mm without ulceration	0	0	IA	IA
T1b	< 0.8 mm with ulceration	0	0	IB	IB
T1b	0.8‒1.0 mm with OR without ulceration	0	0	IB	IB
T2a	> 1.0‒2.0 mm without ulceration	0	0	IB	IB
T2b	> 1.0‒2.0 mm with ulceration	0	0	IIA	IIA
T3a	> 2.0‒4.0 mm without ulceration	0	0	IIA	IIA
T3b	> 2.0‒4.0 mm with ulceration	0	0	IIB	IIB
T4a	> 4.0 mm without ulceration	0	0	IIB	IIB
T4b	> 4.0 mm with ulceration	0	0	IIC	IIC

T, Definition of the primary tumor; N, Characteristics of regional lymph nodes; M, Distant metastasis. Reprinted with permission and adapted from Gershenwald JE, Scolyer RA, Hess KR, Sondak VK, Long GV, Ross MI, et al. Melanoma staging: Evidence-based changes in the American Joint Committee on Cancer eighth edition cancer staging manual. CA, A Cancer Journal for Clinicians. 2017;67:472-92.^30^

The term TM is historically used in the literature and in research and comprises tumors that have an IB ≤ 1.0 mm. Until 2002, the AJCC defined TM as lesions ≤0.76 mm, and the changes in this definition and in the staging over the years make it difficult for meta-analysis studies and often cannot be compared with present data.^72^

Currently, there is no recommendation for using neoadjuvant or adjuvant treatment for MIS and TM. If these patients progress to metastatic/advanced stages, they can benefit from anti PD-1 (pembrolizumab and nivolumab), anti CTLA-4 (ipilimumab); and/or mutation-directed therapies (dabrafenib/trametinib, vemurafenib/cobimetinib encorafenib/binimetinib), with well-established results, according to their staging.^46^

Radiotherapy remains indicated for palliative treatments or in inoperable cases, for local control of the disease. Use of topical medications should be restricted to exceptional situations and/or palliative cases in whom resection is not feasible or desirable. Topical Imiquimod (IMQ) has been used for MIS, particularly LM, as a first-line, second-line, or adjuvant therapy, with high rates of clinical and histopathological clearance. Patient response to topical medication can vary, and there is a need for long-term studies to further validate its efficacy. Therefore, the decision to use IMQ should be made collaboratively with the patient and discussed in tumor boards, in cases where surgery is not viable.^46^

A thorough history and clinical examination, including not only the area of the melanoma scar for the detection of LR, but also the entire body surface, with dermoscopy performed on all pigmented and non-pigmented lesions, is required. Total body digital dermoscopy can aid patient surveillance for new skin neoplasms.^73^

LN palpation is mandatory. Imaging should be performed based on specific patient signs and symptoms.^46^ LN Doppler ultrasound can assist dermatologists in assessing patients with challenging physical examinations (e.g., obesity, inguinal folds) when performed by a trained and experienced specialist. Performing a high-quality clinical examination is paramount, highlighting the dermatologist's role in the follow-up of melanoma patients, since they are at a higher risk of developing new melanomas than metastases.^46^, ^62^, ^65^, ^66^

There is no need for baseline/follow-up laboratory tests or imaging in MIS and TM; they should be considered in patients with a Breslow >0.8 mm. Clinical follow-up aims at early detection of recurrence, subsequent primary melanomas, and education.^46^

Patient education regarding SCC, BCC and new melanomas can aid early diagnosis and modify personal risk factors (Fig. 2). This should be tailored to the patient's educational level in simple language and focused on patient counseling. MIS and TM patients should not be discharged since they have an increased risk of new neoplasms and of LR and metastasis.

There are some global discrepancies on how often MIS and TM patients should be followed. Usually, a dermatological consultation every 4-months in the first year of diagnosis, followed by every 6-months in the second year and annually after is sufficient for most patients. The number of visitations can be modified due to patient risk and prognosis factors or due to public health-specific guidelines in each country.^62^, ^65^, ^66^, ^74^^,^^75^

The first five years of follow-up are important because about 90% of metastases occur during this period, with almost two-thirds occurring in the first two years.^65^, ^76^ The risk of late metastasis and recurrence should be kept in mind, so that if they occur, appropriate treatment is not delayed.

Despite advances in recent years, treatment is still challenging in patients with metastatic disease, having a high mortality rate when diagnosed in advanced stages. Thus, the measure with the greatest impact to reduce mortality is based on early detection of initial tumors, maximizing survival rates.^5^, ^60^

### Local recurrence and metastasis

Early diagnosis and appropriate surgical treatment for MIS and TM are the most important factors in patient survival. Despite their good prognosis, since this is the largest number of melanoma patients, a significant number of patients will have LR or metastasis, and this should be promptly diagnosed by dermatologists.

There is a lack of uniformity in the definition of LR in the literature. Most authors consider LR to be the reappearance of the tumor in the scar or adjacent to the initial surgical procedure. Some studies use the nomenclature, distant recurrence, for LN involvement or metastasis.^76^, ^77^, ^78^, ^79^

The rate of LR in MIS is variable, ranging from 0.3 to 9%, with most studies having a small number of individuals. In TM, the LR rate ranges from 2% to 11.3%, depending on the study design and follow-up time. Table 4 summarizes the findings of LR and metastasis in MIS and TM in the literature.^25^, ^80^, ^81^, ^82^, ^83^, ^84^, ^85^, ^86^

**Table 4:** Studies that evaluated local recurrence and metastasis in early melanomas.

Author	Time period	Number of individuals – n (staging included)	Recurrence rate	Metastasis rate	Average follow-up time	Survival
Gontijo et al.^25^	1997‒2020	1122 (580 MIS and 542 T M)	2.4% MIS; 1.3% TM	0.3% MIS; 2.2% TM	79.5 months MIS; 77.1 months TM	
Gimotty et al.^88^	1988‒2002	26736 (TM)		1.,60%	97 months	89.1% to 99% (20 years)
Leiter et al.^92^	1976‒2007	23842 (stage I)	7.1%		53 months	89% (no recurrence at 10 years)
Lamb et al.^93^	1980‒2015	10928 (TM)		4.5% (LN only)		
Claeson et al.^32^	1995 - 2014	1613 (TM)		1,5%		
Kunishige et al.^82^	1982‒2008	1072 (MIS)	0.30%	0.2%	56 months	
Durham et al.^89^	2005‒2015	512 (0.75 to 0.99 mm)		6.8%	48 months (average)	
Hou et al.^94^	1995‒2005	407 (LM)	4.49%	0		7.9 years
Joyce et al.^81^	2008‒2014	410 (MIS)	2.20%	0.24%	26 months	
Bricca et al.^87^	1980‒2002	331 (MIS) and 294 (invasive)	0	0.7% MIS and 1.9% TM	58 months	99.2% for MIS and 100% TM (5 years)
Akhtar et al.^84^	2001‒2009	192 (MIS)	2.9%	0	31 months	
Bene et al.^83^	12 years	167 (MIS)	1.8%	0	63 months	
Huilgol et al.^80^	1993‒2002	165 (TM and MIS)	2%		38 months	
Murali et al.^95^	1983‒2003	178 (with metastasis) and 178 control	3.20%		79 months	
Moura et al.^85^	2009‒2014	155	9%	1.8%	36 months	
Nosrati et al.^86^	1978‒2015	662	4.07%			92%‒94% (5 years)

LN, Lymph Node; LM, Lentigo Maligna; TM, Thin Melanoma; MIS, Melanoma in situ.

**Fig. 4:**
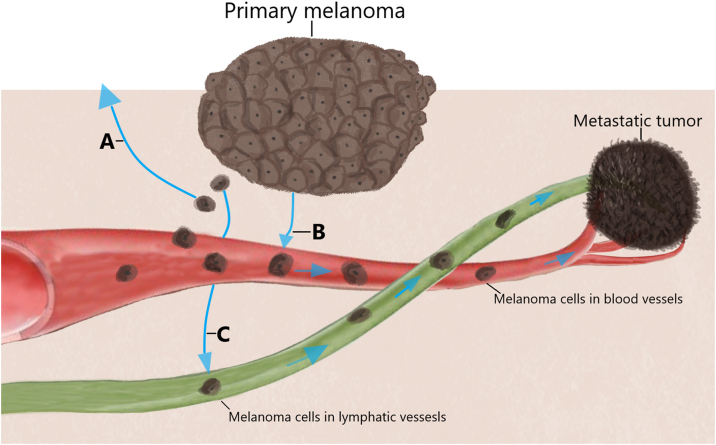
Melanoma dissemination pathways. Tumor cells can spread through adjacent skin (A), blood vessels (B) and lymphatic vessels (C). Its dissemination can occur either simultaneously or individually and lead to local recurrence, satellitosis and distant metastasis.

Metastases are defined as invasion of the tumor into an organ or tissue, with melanoma being a neoplasm with lymphatic and hematogenous dissemination (Fig. 4). It is estimated that in up to two-thirds of cases, they are locoregional, affecting the skin or adjacent lymphatic system.^76^ In almost half of metastatic melanomas, only one organ is affected, with the skin accounting for about 20% of cases and the lungs, liver, and brain for 50%.^37^

These regional metastases can be classified as satellitosis, in transit, or nodal (LN involvement), according to the distance from the primary tumor.^37^ Satellitoses are metastatic papules/nodules that appear within 2 cm of the primary tumor. They may be adjacent to the surgical scar, and their differential clinical diagnosis with LR may be difficult, and it is necessary to highlight their dermal component on histology. In-transit metastases represent invasion of the tumor into the skin or subcutaneous tissue and are located 2 cm beyond the primary site and the LN drainage.^37^ Nodal metastases are more common at the nodal draining site of the primary tumor; nevertheless, they can also be found in discordant and unexpected LN drainages. In about 3% of patients with metastases, the primary site is not found.^65^

Presence of metastasis during follow-up of MIS patients raises the question about primary tumor depth missed by the pathologist and possible presence of another thick or unknown melanoma that may be the origin of metastatic disease. Studies reporting metastasis in this group are scarce, with rates ranging from 0.24% to 1.8%.^25^, ^81^, ^85^, ^87^

The rate of metastasis in patients with TM is rarely reported; if the authors exclude studies of LN involvement, being from 1.6% to 6.8%, according to Breslow thickness and study design.^25^, ^77^, ^88^, ^89^

Late recurrence is generally defined by most studies as the recrudescence of the disease after 10-years (some authors consider it to be late after 5-years) and early recurrence when it occurs before this period. Late recurrence incidence can reach up to 6.9%, varying according to the population studied.^78^, ^79^ These data show that patients with melanoma have a rate of LR even after long follow-up periods.

Some authors state that melanomas may possibly remain quiescent for decades in individuals until the development of LR or metastasis. In an analysis of 2,766 melanomas staged I‒IV, from 1960 to 1996, Tsao et al. found a 18.1-years period for regional recurrence and 19-years for distant metastasis, showing that ultra-late recurrence (more than 15-years after diagnosis) can occur and without identifiable risk factors in the study.^79^

Progression rate from MIS to invasive melanoma is not known, but the rarity of LR and the exceptional deaths of these patients due to metastases suggest that not all MIS lesions would be precursors of invasive tumors and may remain without vertical or invasive growth. MIS can be considered a risk factor for the development of a second melanoma, which may present aggressive and invasive behavior.^7^

A limitation of long-term studies is that patients who died from causes other than melanoma may have died from occult metastasis and are not accounted. The incidence of occult metastasis can only be verified through autopsy, a procedure that is difficult to access and in some countries is subject to strict legal requirements.

Another possible bias when interpreting LR and metastasis is the lack of uniformity in large databases. A study of SEER data revealed that in one data center, a quarter of TM had Breslow depth errors. These tumors were reclassified as Breslow >1.0 mm, including 96% of the deaths associated with TM.^90^

When diagnosed in early stages (T1a), patients have a five-year survival rate of 99% and a 10-year survival rate of 98%. As these tumors progress, 5-year survival drops to 82% and 10-year survival to 75% in T4b N0 patients.

Since MIS and TM represent up to 83% of new melanoma diagnoses, even a 2% lethality rate represents a massive number of patients dying from early-stage disease, currently representing more than 30% of all melanoma deaths.^9^ In Australia, there are currently more deaths related to TM than to thick melanomas; these tumors comprise a substantial fraction of the overall burden of lethal melanomas in this high-incidence population.^91^

Careful consideration must be taken when advising MIS and TM patients on their diagnosis, follow-up and risk factors. One should not state that MIS or TM patients are disease-free (“cured”) and do not require follow-up. Strong current data prove that these patients have a risk of LR and metastasis and will most likely develop a secondary melanoma, BCC, or SCC. Current prognostic tools do not allow us to stratify which patients are at higher risk for a worse outcome. Since initial melanomas are the majority of melanoma diagnoses, this gives dermatology an opportunity for patient education, screening, and facilitating secondary prevention. Aggressive behavior towards MIS and TM with expensive imaging and exams might also not be the correct approach for a vast number of patients, placing an economic and psychological burden on patients.

## Conclusion

MIS and TM have a growing incidence and importance, representing a substantial part of dermatological practice. Individual risk stratification, early diagnosis, and patient information about prevention are essential to reducing incidence and mortality. Strict clinical follow-up will facilitate timely diagnosis of recurrences, metastases, secondary melanomas, and other skin neoplasms, reinforcing the need for continuous long-term follow-up of these patients. New treatments and diagnostic tools will possibly be incorporated into the future management of these patients by dermatologists, making it essential to update them.

## ORCID ID

Flávia Vasques Bittencourt: 0000-0003-0087-6806

Jacob H. Nelson: 0000-0002-6336-4210

Lucas Campos Garcia: 0000-0002-7883-5986

Sancy A. Leachman: 0000-0001-9140-0648

## Financial support

None declared.

## Authors' contributions

João Renato Vianna Gontijo: Writing of the article; critical review of the important intellectual content and final approval of the final version of the manuscript.

Flávia Vasques Bittencourt: Writing of the article; critical review of the important intellectual content and final approval of the final version of the manuscript.

Jacob H. Nelson: Writing the article; critical review of the important intellectual content, and final approval of the final version of the manuscript.

Lucas Campos Garcia: Writing the article; critical review of the important intellectual content, and final approval of the final version of the manuscript.

Sancy A. Leachman: Writing the article; critical review of the important intellectual content, and final approval of the final version of the manuscript.

## Research data availability

The entire dataset supporting the results of this study was published in this article.

## Conflicts of interest

None declared.
